# Pioglitazone Modifies Kupffer Cell Function and Protects against *Escherichia coli*-Induced Bacteremia in Burned Mice

**DOI:** 10.3390/ijms232112746

**Published:** 2022-10-22

**Authors:** Hiromi Miyazaki, Manabu Kinoshita, Hiroyuki Nakashima, Shingo Nakamura, Daizoh Saitoh

**Affiliations:** 1 Division of Biomedical Engineering, National Defense Medical College Research Institute, Saitama 359-8513, Japan; 2 Department of Immunology and Microbiology, National Defense Medical College, Saitama 359-8513, Japan; 3 Division of Traumatology, National Defense Medical College Research Institute, Saitama 359-8513, Japan

**Keywords:** burns, Kupffer cells, PPARγ, infection, phagocytosis, bacterial clearance

## Abstract

Infectious complications and subsequent sepsis in severely burned patients lead to high morbidity and mortality in response to uncontrolled innate immune responses mediated by macrophages. Peroxisome proliferator-activated receptor gamma (PPARγ) has anti-inflammatory activity and acts as a master regulator of macrophage polarization. In this study, we investigated whether the administration of a PPARγ agonist could modulate the Kupffer cell phenotype and thereby ameliorate the dysregulated innate response during post-burn bacterial infection. C57BL/6 mice were subjected to severe burns and randomized to receive either the PPARγ agonist, pioglitazone, or the vehicle control five days after injury, followed by the subsequent analysis of hepatic macrophages. Survival from the bacterial infection was monitored for seven days. Pioglitazone protected burned mice against bacterial infection. A single treatment with pioglitazone significantly enhanced phagocytosis, phagosome acidification, bacterial clearance, and reduction in inflammatory mediators in Kupffer cells. In conclusion, PPARγ activation by pioglitazone prevents clinical deterioration due to post-burn bacterial infection and improves survival. Our findings suggest that pioglitazone may be an effective therapeutic candidate for post-burn infectious complications.

## 1. Introduction

Infection is one of the most common and serious complications in burn patients [1]. In general, burn wounds, the respiratory tract, urinary tract, and bloodstream are the most frequent sites of infection in severely burned patients. Increase in the total body surface area and depth of burns correlate with an excessive risk of infectious complications [2]. Despite the widespread use of antibiotics and advanced supportive care, infectious complications are now the main cause of death in patients with severe burns. Previous studies have shown that 34–65% of deaths in burn patients are attributed to infectious complications or sepsis [1,3,4,5]. The mortality rates in septic burn patients associated with infectious complications remain high, and fighting such infections is a real challenge [3]. With the growing threat of multidrug-resistant pathogens in burn injuries [1], new strategies, apart from antibiotic therapy, are required to prevent or treat these infections.

During sepsis, the liver plays an essential role in the defense responses against microorganisms, such as bacterial clearance, cytokine production, and metabolic adaptation to inflammation. Hepatic macrophages, consisting of Kupffer cells (KCs) and recruited monocyte-derived macrophages, are the largest population of innate immune cells in the liver that play a central role in maintaining liver homeostasis [6]. In particular, KCs constitute ~90% of total tissue macrophages in the body [7], effectively capturing and eliminating the blood-borne bacteria. Phagocytosis and killing of microbial pathogens are critical components of the early phase of host defense against bacterial infections [8,9]. However, macrophages act as double-edged swords, as their hyperactivation can lead to increased inflammation and contribute to the progression of organ injury [6]. Severe burn injury induces the activation of an inflammatory cascade in various organs, including the liver, via the recruitment of monocytes and activated macrophages. Its activation further leads to the development of immunosuppression, increased susceptibility to sepsis, and multiple organ failure. Previous studies by our group [10,11,12] and others [13] have demonstrated that tissue macrophage dysfunction can lead to immunoparalysis and immunosuppression, associated with increased susceptibility to life-threatening infections. Thus, the adequate immunomodulation of liver macrophages may be an effective therapeutic target for controlling infections in severely burned patients.

Peroxisome proliferator-activated receptor gamma (PPARγ) is expressed in various immune cells, including macrophages, and regulates metabolic and inflammatory signaling pathways [14,15]. PPARγ plays an important role in regulating inflammation and host immune responses [16,17]. Indeed, several animal studies have reported that targeting PPARγ activity with the specific agonist, pioglitazone, may be beneficial and improve the inflammation-related immune dysfunction or morbidity via anti-inflammatory and immunomodulatory effects [18,19,20,21,22]. PPARγ also plays an important role in modulating macrophage polarization [15,23]. Macrophage polarization is a key mechanism for regulating the inflammatory response, and an imbalance in macrophage M1/M2 polarization is often associated with various diseases and inflammatory conditions [23]. Several studies have shown that the manipulation of PPARγ activity has the potential to balance M1/M2 macrophage polarization and prevent the development of organ disorders and local pro-inflammatory response [24,25,26,27].

Therefore, we believe that immunotherapies aimed at augmenting the host resistance to infection, such as improving phagocyte function after burn injury, can be efficacious in this setting. Thus, this study investigated whether the PPARγ agonist, pioglitazone, can protect against post-burn bacterial infection and determined the effects of PPARγ activation in mouse models.

## 2. Results

### 2.1. PPARγ Activation Protects Mice against Postburn Bacterial Infection

**Figure 1:**
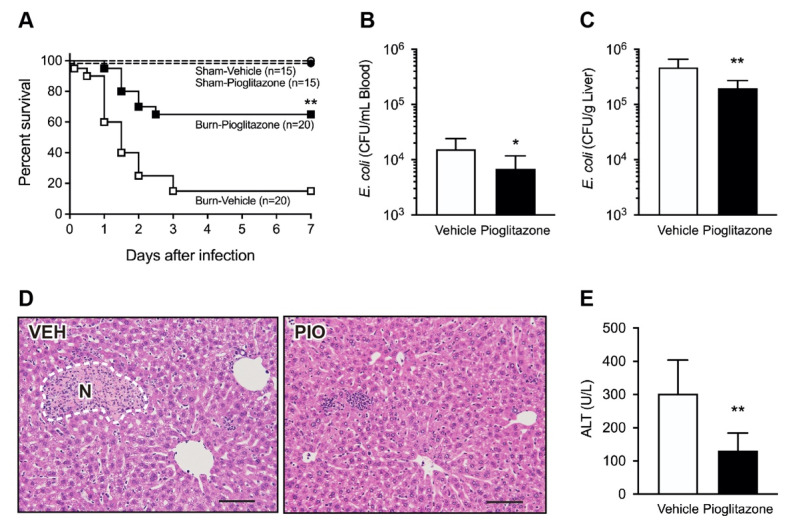
Pioglitazone improves bacterial clearance and survival in post-burn *Escherichia coli*-infected mice. Mice were treated with pioglitazone or vehicle control intraperitoneally 3 h before bacterial challenge and five days after burn injury. (**A**) Survival of mice after intravenous infection with *E. coli* (5 × 10^8^ CFU). Mice were monitored every 12 h for the first three days and then once per day until seven days after infection. Sham: *n* = 15 per group, Burn: *n* = 20 per group, vehicle- or pioglitazone-treated mice; pooled data of two independent experiments. (**B**–**E**) To determine systemic bacterial clearance, mice (*n* = 8 per group) were intravenously injected with *E. coli* (1 × 10^8^ CFU), and the blood and liver were harvested aseptically 24 h after infection. Bacterial numbers in the blood (**B**) and liver (**C**) of the vehicle or pioglitazone-treated mice. (**D**) Representative hematoxylin and eosin staining of liver tissues 24 h after infection (200× magnification). Dashed line indicates the border of altered tissue forming a focal necrotic area (N). Bars = 50 μm. (**E**) Serum levels of alanine transaminase (ALT) 24 h after infection. CFU, colony forming unit. Data are presented as the mean ± standard deviation (SD); * *p* < 0.05; ** *p* < 0.01 vs. vehicle.

To evaluate the effects of pioglitazone treatment on post-burn bacterial infection, burned and sham mice were injected with the Gram-negative bacterium, *Escherichia coli*, on day 5 after injury. Mice were treated with pioglitazone or vehicle control via intraperitoneal injection 3 h before bacterial challenge. In response to infection, 50% of the burned mice treated with the vehicle succumbed to fatal shock within 24 h, and 85% of the animals died within three days. In contrast, 65% of burned mice treated with pioglitazone survived after seven days. The survival rate of pioglitazone-treated mice was significantly higher than that of vehicle-treated mice (Figure 1A). In contrast, all survived unburned sham mice survived, with no adverse symptoms, regardless of the treatment groups.

To further elaborate on the mechanism underlying the increased survival of pioglitazone-treated mice, we confirmed the bacterial burden in the peripheral blood and liver 24 h after infection. In line with the observation of the mortality rate, pioglitazone treatment significantly decreased the bacterial load in both the blood (Figure 1B) and liver (Figure 1C). In addition, histopathological examination revealed numerous focal loci of necrotic hepatocytes in the livers of vehicle-treated mice. In contrast, the administration of pioglitazone attenuated the degenerative changes and decreased the levels of alanine transaminase (ALT), indicative of hepatocyte necrosis (Figure 1D,E).

**Figure 2:**
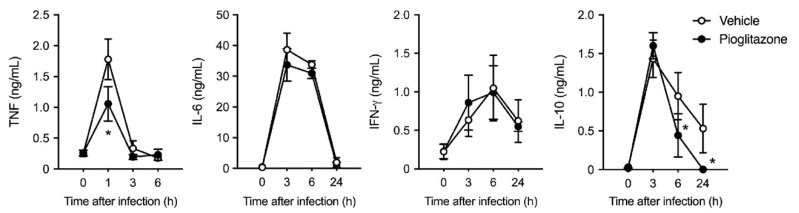
Pioglitazone attenuates systemic inflammatory responses in post-burn-infected mice. Tumor necrosis factor (TNF), interleukin (IL)-6, interferon (IFN)-γ, and IL-10 levels were measured using enzyme-linked immunosorbent assay (ELISA) in serum samples collected at the indicated time points after bacterial infection. *n* = 8 per group; pooled data of two independent experiments. Data are represented as the mean ± SD; * *p* < 0.05 vs. vehicle.

Since the excessive production of cytokines plays a vital role in septic shock and mortality, we measured the serum cytokine levels up to 24 h after *E. coli* infection. Although the levels of serum cytokines (tumor necrosis factor [TNF], interleukin [IL]-6, interferon [IFN]-γ, and IL-10) were drastically increased in burn-injured mice under sepsis, pioglitazone reduced the peak level of TNF and continuous production of IL-10 after infection. In contrast, IL-6 and IFN-γ levels were comparable in both groups at different time points (Figure 2).

**Figure 3:**
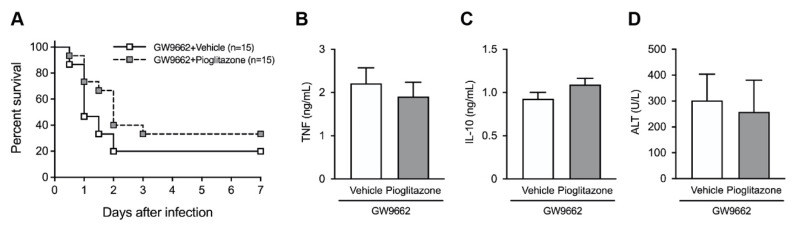
Peroxisome proliferator-activated receptor gamma (PPARγ) antagonist, GW9662, abolishes the protective effects of pioglitazone in post-burn infection. (**A**) Survival of burned mice after intravenous infection with *E. coli* (5 × 10^8^ CFU). Serum concentration of TNF at 1 h (**B**), IL-10 at 6 h (**C**), and ALT at 24 h (**D**) after infection. *n* = 15 per group; pooled data of two independent experiments. Data are represented as the mean ± SD.

We examined whether the pharmacological blockage of PPARγ activation abrogated the favorable effect of pioglitazone using the PPARγ antagonist, GW9662. Burn-injured mice were pretreated with GW9662 30 min before the pioglitazone challenge. Pretreatment with GW9662 did not alter the survival rate, serum cytokine levels, or liver damage in response to *E. coli* infection in vehicle- or pioglitazone-challenged mice (Figure 3A–D). The inhibition of PPARγ activation abrogated the protective effects of pioglitazone in post-burn infections.

### 2.2. Pioglitazone Promotes the Recruitment of Inflammatory Monocyte/Macrophages into the Liver

**Figure 4:**
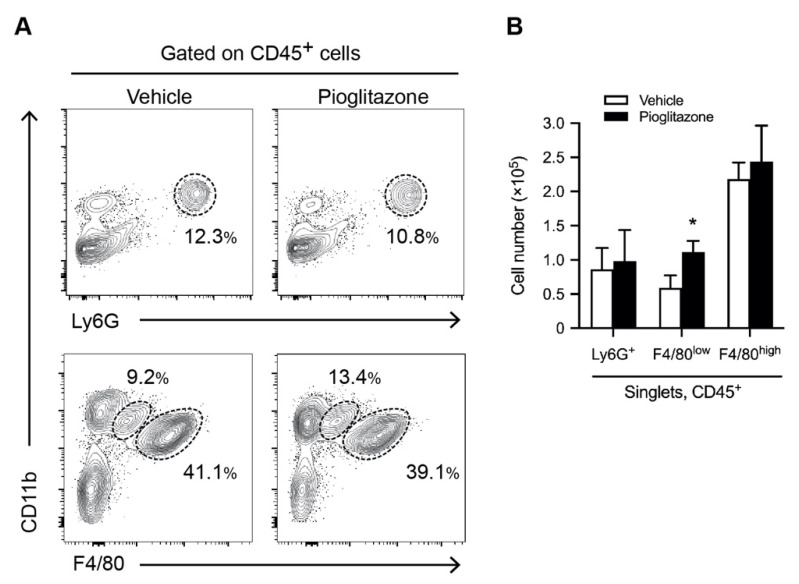
Pioglitazone treatment increases the number of CD11b^+^ F4/80^low^ monocytes/macrophages in burned mice. Five days after burn injury, hepatic immune cells were obtained from mice 3 h after treatment with vehicle or pioglitazone. Isolated whole liver mononuclear cells (MNCs) were subjected to flow cytometry analyses. FACS plots and cell numbers of neutrophils (CD11b^+^ Ly6G^+^), recruited monocytes/macrophages (CD11b^+^ F4/80^low^), and Kupffer cells (KCs) (CD11b^+^ F4/80^high^) are shown in CD45^+^ gated whole liver MNCs isolated from burned mice with vehicle or pioglitazone treatment. Representative FACS plots (**A**) and a graphical summary (**B**) showing the cell number of indicated liver immune cell subsets. *n* = 6 per group. Data are represented as the mean ± SD; * *p* < 0.05 vs. vehicle.

To determine the population of myeloid cells and resident macrophage KCs following pioglitazone treatment, we analyzed the phenotype of hepatic leukocytes isolated from mice with vehicle and pioglitazone treatment five days after burns using flow cytometry. We assessed CD11b and F4/80 expression levels in CD45^+^ cells based on prior data that resident KCs were CD11b^+^ F4/80^high^, whereas infiltrating monocytes/macrophages were CD11b^+^ F4/80^low^. CD11b^+^ Ly6G^+^ cells were defined as neutrophils. Our data showed that the number of CD11b^+^ F4/80^low^ monocytes/macrophages within the CD45^+^ liver cell population was increased in pioglitazone-treated mice. In contrast, the numbers of CD11b^+^ Ly6G^+^ neutrophils and CD11b^+^ F4/80^high^ KCs did not change in the pioglitazone-treated mice (Figure 4).

### 2.3. Treatment with Pioglitazone Enhances Phagocytosis and Bactericidal Activity in Burned Mice

**Figure 5:**
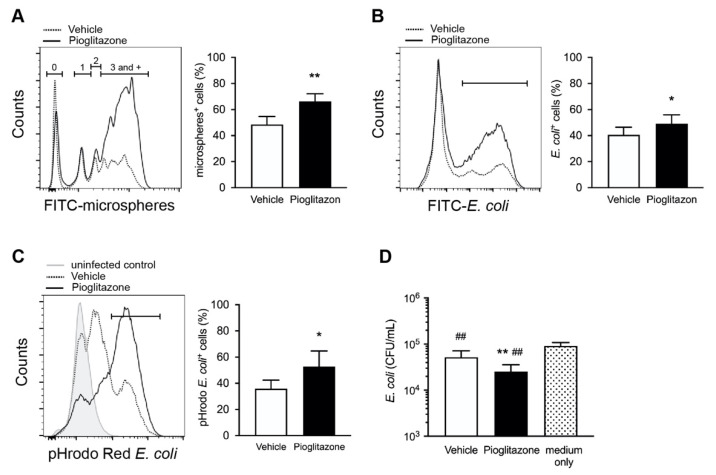
Pioglitazone treatment enhances bacterial clearance in burn-injured mice. (**A**,**B**) Isolated hepatic immune cells 3 h after treatment were incubated with fluorescein-conjugated microspheres or *E. coli* for 30 min and stained with anti-CD45, anti-CD11b, and anti-F4/80 antibodies, followed by the analysis of phagocytic activity in CD11b^+^ F4/80^high^ KCs via flow cytometry. (**A**) Representative flow cytometry histogram showing uptake of FITC-microsphere by KCs from vehicle and pioglitazone-treated mice after burns. Each peak represents the KC population that has ingested either 0, 1, 2, 3, or more microspheres. Percentage of KCs which has internalized ≥ three microspheres was determined. (**B**) Representative histograms showing phagocytosis of FITC-conjugated *E. coli* by KCs and percentage of FITC-conjugated *E. coli* phagocytosing KCs. (**C**) Representative flow cytometry histogram showing a shift in fluorescence intensity of pHrodo *E. coli* bioparticles in CD11b^+^ F4/80^high^ KCs. The pHrodo fluorescence increased with decreasing pH (acidification), indicating phagosomal maturity after ingested bioparticles. Percentage of *E. coli* particle-positive KCs with low pH phagosome in vehicle and pioglitazone-treated mice 20 min post administration of pHrodo *E. coli*. *n* = 6 per group. (**D**) In vitro bacterial killing of hepatic immune cells from vehicle or pioglitazone-treated mice. Isolated cells were incubated with *E. coli* for six hours. As a control, *E. coli* were incubated without immune cells in the culture medium. *n* = 6 per group; Data are represented as the mean ± SD; * *p* < 0.05; ** *p* < 0.01 vs. vehicle. ## *p* < 0.01 vs. medium only.

Macrophages are phagocytes that play a critical role in the host defense against bacteria. Because KCs severely impair phagocytic activity after burns [10,12], we next investigated whether pioglitazone treatment influenced phagocytosis and bactericidal activity. The phagocytic activity of CD11b^+^ F4/80^high^ KCs in vitro was assessed using flow cytometry with fluorescence-conjugated microspheres. Based on our previous studies [10,12], we defined the metrics of uptake capacity as the proportion of highly phagocytic cells (peak ≥3). Compared with vehicle-treated KCs, pioglitazone significantly augmented the phagocytic responses in microsphere phagocytosis assay (Figure 5A). We also assessed the phagocytic function of KCs in response to *E. coli* infection. As expected, we observed an increase in FITC-conjugated *E. coli* phagocytosis in the KCs of pioglitazone-treated mice after injury compared to that in vehicle-treated mice (Figure 5B).

To measure alterations in the bactericidal capacity of KCs in vivo, we examined phagosome acidification in KCs engulfed with *E. coli* using pH-sensitive pHrodo-conjugated bacteria. Phagosomal acidification is required for phagolysosome formation and supports the bactericidal activity of macrophages at low pH. Although the acidification of pHrodo Red *E. coli* by KCs was impaired in vehicle-treated mice, the pioglitazone treatment increased the proportion of KCs that acidified the phagosome (Figure 5C). To test the hypothesis that upregulated phagocyte function was responsible for the decreased bacterial burden observed in the liver of pioglitazone-treated mice (Figure 1C), we incubated *E. coli* with hepatic immune cells, including KCs. Viable bacteria were counted by culturing them on Luria–Bertani (LB) agar. Concurrent with increased phagocytosis, immune cells from pioglitazone-treated mice were characterized by a decrease in the number of bacteria compared to that of the control (Figure 5D), which indicates boosted bactericidal activity with treatment.

In this study, the CD11b^+^ F4/80^low^ subset contained two populations, one of which was a non-phagocyte monocyte, whereas the other was a phagocyte macrophage. CD11b^+^ F4/80^low^ infiltrated monocytes/macrophages expanded with pioglitazone treatment (Figure 4); there were no appreciable differences in the phagocytic and bactericidal activity across the treatment groups (Figure S1A–C in Supplementary Material).

### 2.4. Pioglitazone Directs Liver F4/80^+^ Cells toward M2-like Phenotype in Burn-Injured Mice

**Figure 6:**
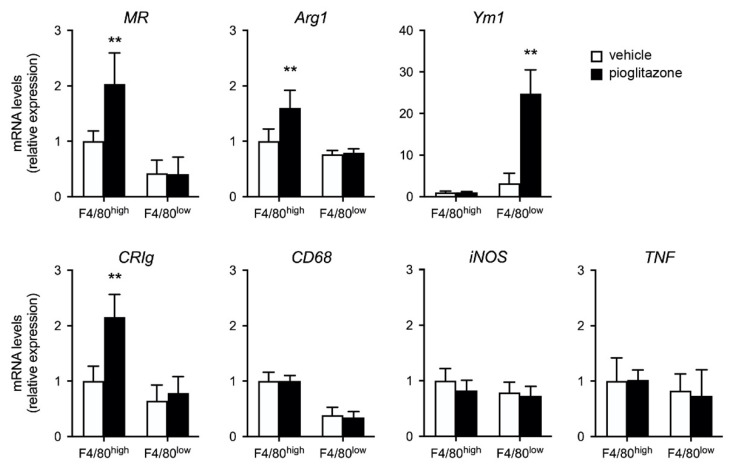
Pioglitazone induces M2 markers and *CRIg* in liver F4/80^+^ cells after burn injury in vivo. Gene expression changes in F4/80^high^ KCs and F4/80^low^ recruited (infiltrated) monocytes/macrophages. Five days after burn injury, indicated cell types were harvested from the vehicle and pioglitazone-treated mice 3 h after treatment and subjected to quantitative reverse transcription-polymerase chain reaction (qRT-PCR) analysis to determine the expression levels of macrophages M1/M2 markers. All values were normalized for ribosomal protein S18 (*Rps18*) and are presented relative to F4/80^high^ KCs from vehicle-treated mice. *n* = 4 per group. Data are represented as the mean ± SD; ** *p* < 0.01 vs. vehicle.

Previous studies have demonstrated that PPARγ activation promotes a shift from M1 to M2 macrophages and modulates macrophage function [14,16]. Therefore, we performed M1/M2 marker RNA analysis of F4/80^high^ and F4/80^low^ cells isolated from vehicle- and pioglitazone-treated mice following burn injury. The expression levels of genes encoding arginase 1 (*Arg1*) and mannose receptor (*MR*; *CD206*), the hallmarks of M2 macrophages, were higher in F4/80^high^ KCs, but not F4/80^low^ cells, obtained from pioglitazone-treated mice than in vehicle-treated mice. The levels of chitinase-like protein 3 (*Chil3*; *Ym1*), which is expressed in myeloid cell lineages and used as a marker of activated M2 macrophages, were considerably higher in pioglitazone-elicited F4/80^low^ cells than in vehicle-treated cells. Interestingly, the levels of a complement receptor, *CRIg*, uniquely expressed in tissue-resident macrophages, were augmented in pioglitazone-treated F4/80^high^ KCs. In contrast, the expression levels of the macrophage markers, *CD68*, and inducible nitric oxide synthase (*iNOS*) and *TNF* (M1 markers), were not altered in F4/80^high^ or F4/80^low^ cells after treatment (Figure 6).

## 3. Discussion

Patients with burns have a high prevalence of sepsis and poor outcomes. Our findings showed that PPARγ activation by pioglitazone improved the outcomes of severely burn-injured mice after *E. coli* challenge and sepsis. Here, we describe the effects of PPARγ activation on the regulation of KC inflammatory and phagocyte-bactericidal responses. Gene expression profiling of F4/80^high^ KCs and F4/80^low^ infiltrated monocytes/macrophages in the liver revealed an M2-like profile characterized by increased pathogen capture and anti-inflammatory responses. The administration of a PPARγ antagonist to burned mice abolished the protective effect of pioglitazone against sepsis after bacterial infection. In addition, pioglitazone may have alleviated the early excessive cytokine response after bacterial challenge in burn-injured mice. The beneficial effect of pioglitazone on post-burn infectious complications may synergistically act with enhanced bacterial clearance and reduced uncontrolled inflammatory responses to bacterial challenges.

PPARγ plays an essential role in the immune response by inhibiting the expression of inflammatory cytokines and regulating alternative macrophage activation. Pioglitazone, a United States Food and Drug Administration-approved PPARγ agonist, is widely used to regulate inflammation and macrophage polarization. In cecal ligation and puncture-induced sepsis models [20,28], pioglitazone prevented excessive inflammation and improved sepsis outcomes. Macrophage polarization is a host mechanism that controls the proper direction of immune responses, M1 for the pro-inflammatory and M2 for anti-inflammatory responses [23,29]. Pioglitazone regulates macrophage polarization to M2 subtype. Several studies have shown that the manipulation of PPARγ activity has the potential to balance M1/M2 macrophage polarization and prevent the development of organ disorders and local pro-inflammatory responses [24,25,26,27]. Indeed, our data showed that pioglitazone increased the expression levels of M2 markers, *MR*, *Arg1*, and *Ym1* in liver F4/80^high^ KCs and F4/80^low^ monocytes/macrophages and alleviated the cytokine response to bacteria, supporting the anti-inflammatory effects of PPARγ agonists in previous studies. In general, monocytes recruited to an injured site exhibit a pro-inflammatory phenotype and contribute to the increase in inflammation. The roles of monocyte migration in the resolution of inflammation and tissue repair have been previously reported [30]. Bone marrow-derived monocytes differentiate into Ly6C^high^ and Ly6C^low^ macrophages and exert either pro-inflammatory or anti-inflammatory effects with the assistance of the local environment [31]. Ikeda et al. [32] reported that Ym1^+^ Ly6C^high^ monocytes exhibit immunoregulatory and tissue-reparative phenotypes after infiltrating the injured tissue. Increased CD11b^+^ F4/80^low^ cells in pioglitazone-treated mice showed markedly higher *Ym1* expression than vehicle controls. These results suggest that a distinct monocyte/macrophage subpopulation destined to act in immunoregulation may be induced by pioglitazone and participate in the resolution of inflammation. Differential Ly6C expression can identify functionally distinct macrophage populations. However, in this study, the CD11b^+^ F4/80^low^ subset was a mixture of monocytes and macrophages; therefore, we were unable to distinguish between these cells and evaluate Ly6C expression in F4/80^low^ macrophages. It also remains to be determined whether bone marrow-derived F4/80^low^ cells differentiate into resident KCs in the liver of burned mice.

Since delayed pathogen elimination is the first step in the development of sepsis, bacterial clearance is one of the most crucial processes for patient mortality. Liver-resident macrophages, KCs, have been recognized as the major phagocytes in sinusoids, where they rapidly capture and eliminate most bacteria [7,8,33]. Our data showed that phagocytosis and bacterial killing were significantly augmented by pioglitazone pretreatment in the KCs of burned mice. Phagocytosis of pathogens in the bloodstream depends on opsonins, including antibodies and complement components [33,34]. Several receptor-mediated pathogen-recognition mechanisms are known for the capture of intravascular bacteria by KCs [33,35]. The complement receptor immunoglobulin CRIg is uniquely expressed on KCs and binds to bacterial pathogens in both compliment-dependent and -independent manners, thereby supporting bacterial capture [35,36]. In burned mice, we found that *CRIg* expression on KCs was increased following pioglitazone treatment, which may contribute to the upregulation of opsonized bacterial phagocytosis. *CRIg* expression can be suppressed by various cytokines (TNF, IFN, and IL-6) [37]. As such inflammatory signs are highly prevalent in severe burns [2,38], severe burn-induced negative modulation of *CRIg* may be associated with restricted bacterial phagocytosis.

During host defense, it is crucial to maintain the acidity of the macrophage phagosome for effective bacterial clearance. In the present study, phagosomal acidification was enhanced in resident KCs isolated from pioglitazone-treated mice compared to that in vehicle-treated mice after burn injury. During phagosomal maturation, phagosomes undergo progressive acidification of the lumen, mainly achieved via proton pumping by vacuolar-type H^+^-ATPase (V-ATPase) [39]. M2-like macrophages promptly ensure phagosome acidification to quickly and effectively achieve bactericidal activity, whereas M1-like macrophages rely on the gradual acidification of their phagosomes [40]. M1-like and M2-like macrophages also differ in phagosome metabolic activity [41], and V-ATPase is essential for the polarization of M2 macrophages to suppress the innate immune response [29]. Therefore, to ensure efficient bactericidal activity, it may be essential to regulate the activity of proteins, such as V-ATPase, in the phagosome by switching to M2-like polarized macrophages. Recent studies have demonstrated a link between cellular metabolism and immune cell functions. Impaired metabolism has been reported as the primary cause of macrophage dysfunction [42]. PPARγ modulates glycolysis-gluconeogenesis [15,22]. Our results indicate that PPARγ prevents liver macrophages from polarizing toward the glycolysis-dependent immune reactive state after burn injury, further confirming its anti-inflammatory effect on macrophages.

Remarkably, our results show that the administration of pioglitazone simultaneously exhibits anti-inflammatory and bactericidal activities, with seemingly contradictory effects. The mechanisms underlying these unique actions of pioglitazone remain unclear and require further elucidation. In infectious complications following severe burns, where optimal bacterial clearance and prevention of excessive inflammation are critical for the survival of patients, PPARγ agonists can be used in novel therapeutic strategies apart from antibiotic therapy.

## 4. Materials and Methods

### 4.1. Animal Model of Burn Injury and Pioglitazone Treatment

Male C57BL/6 (total: *n* = 148, body weight: 24.4 ± 1.9 g) mice were purchased from Charles River Laboratories (Yokohama, Japan). Mice were given free access to food and water and acclimatized in a 12 h light/dark cycle under specific pathogen-free conditions for 7–12 days prior to the initiation of experiments. All animal experiments were conducted according to the institutional ethical guidelines for animal experiments of the National Defense Medical College and were approved by the Animal Care and Use Committee of the National Defense Medical College, Saitama (permit number: 17013, 21035).

A murine model of full-thickness burn injury was employed with minor modifications as described previously [10,12]. Eight-to-ten-week-old mice were anesthetized intraperitoneally with a ketamine (50 mg/kg) and xylazine (10 mg/kg) cocktail, and their dorsum was shaved. The mice were then placed in a plastic template (surface area: 25 × 35 mm) that exposed 20% of the total body surface area and subjected to a full-thickness burn injury by pressing a heated brass blade for one second (BN-100; 100 W, tip temperature: 545 °C, Taiyo Electric, Tokyo, Japan). All mice were resuscitated with 1 mL of saline intraperitoneally and placed on warming pads until recovery from anesthesia. Sham animals underwent a sham procedure that included all interventions except for the actual burn injury. Five days after injury, mice were randomized to receive either the PPARγ agonist, pioglitazone (10 mg/kg; Santa Cruz, CA, USA) or vehicle control (10% *v/v* dimethyl sulfoxide in sterile saline) via intraperitoneal injection.

### 4.2. Systemic Bacterial Challenge

For survival experiments, mice (Sham: *n* = 15/group; Burn: *n* = 20/group) were injected intravenously with *E. coli* (strain B, ATCC #23848; 5 × 10^8^ CFU) 3 h after the administration of vehicle or pioglitazone, and survival rates were monitored every 12 h for the first three days and then once per day until seven days after infection. To alleviate pain or distress, mice were euthanized when a loss of more than 20% of baseline body weight occurred, or the following qualitative humane endpoint criteria were observed during inspection: complete paralysis with the absence of spontaneous movement, severe ataxia or loss of postural reflexes, severe reduction of general health status with reduced grooming, or refusal of food intake. Additionally, to assess the inhibition of PPAR activation, burned mice were intraperitoneally injected with the PPARγ antagonist, GW9662 (2 mg/kg; Cayman Chemical, Ann Arbor, MI, USA) 30 min prior to pioglitazone and vehicle treatment, and mice were subjected to *E. coli* challenge (15 mice/group).

To determine systemic bacterial clearance, mice (8 mice/group) were intravenously injected with *E. coli* (1 × 10^8^ CFU), and the blood and liver were harvested aseptically 24 h after infection. To evaluate the bacterial burden, we quantified the bacterial count in the blood and liver, as previously described [10]. Briefly, the liver samples were homogenized in ice-cold phosphate-buffered saline (PBS). Liver homogenates and blood were serially diluted in sterile PBS and plated onto LB agar plates. The bacterial colony-forming units were counted after 18 h incubation at 37 °C. For general histology, formalin-fixed liver tissues were embedded in paraffin, and 5-μm thick paraffin sections were stained with hematoxylin and eosin.

### 4.3. Isolation of Hepatic Immune Cells Including KCs

Five days after burn injury, hepatic immune cells were obtained from burn-injured mice 3 h after treatment with vehicle or pioglitazone (6 mice/group). As previously described [10,12], mice were perfused with PBS (10 mL) to remove blood from their organs, and livers were collected, minced with scissors, and digested for 20 min at 37 °C in an HBSS solution containing 0.5 mg/mL collagenase type IV (Wako, Osaka, Japan). Digested livers were passed through a 50-μm stainless steel mesh. After washing homogenates in the Roswell Park Memorial Institute (RPMI)-1640 medium containing 10% fetal bovine serum (FBS), hepatocytes and debris were removed via a Percoll gradient. After red blood cells were lysed, the harvested total hepatic immune cells were stored for further analysis.

### 4.4. Flow Cytometry

Non-specific binding was blocked with CD16/32 (93) monoclonal antibody for 15 min at 4 °C, and the cells were stained with the following fluorochrome-conjugated anti-mouse antibodies for 20 min at 4 °C: F4/80-APC (BM8), CD11b-FITC (M1/70), Ly6G-PE (1A8), and CD45-APCeFluor780 (30-F11) (all from eBiosciences, San Diego, CA, USA). Multiparameter acquisition was performed using Cytomics FC500 (Beckman Coulter, Indianapolis, IN, USA), and F4/80^high^ or F4/80^low^ cells were isolated using a cell sorting system (SH800; Sony Corporation, Tokyo, Japan). Flow cytometric analysis was performed using FlowJo 10 software (BD Biosciences, San Diego, CA, USA).

### 4.5. Phagocytosis, Bactericidal Activity, and In Vitro Killing Assays

To assess the phagocytic activity of CD11b^+^ F4/80^high^ KCs, Fluoresbrite YG microspheres (1 μm; Polysciences, Eppelheim, Germany) and FITC-conjugated *E. coli* bioparticles (Thermo Fisher Scientific, Waltham, MA, USA) were used. Isolated hepatic immune cells (5 × 10^5^ cells) were incubated with microspheres (1 × 10^7^ particles) or *E. coli* (1 × 10^6^ particles) for 30 min at 37 °C in 5% CO_2_. Cells were then incubated with Fc-blocker and stained with macrophage markers, and the uptake of each particle by CD11b^+^ F4/80^high^ KCs was assessed via flow cytometry. Before the experiments, *E. coli* bioparticles were opsonized using a BioParticles opsonizing reagent (Molecular Probes, Eugene, OR, USA).

For in vivo bactericidal ability, mice were injected intravenously with pH-sensitive pHrodo-Red *E. coli* bioparticles (1 × 10^7^ particles; Thermo Fisher Scientific). After 20 min, the livers were collected and immune cells, including KCs, were harvested using the above-mentioned methods. The dye is non-fluorescent at neutral pH and exhibits bright red fluorescence at acidic pH. The advantage of this is that actual phagocytosis and phagosomal acidification can be measured, whereas extracellular adherent particles can be left undetected. We determined phagosome acidification in KC-engulfed *E. coli* bioparticles using flow cytometry.

To assess intracellular bacterial killing in vitro, isolated hepatic immune cells (5 × 10^5^ cells) were resuspended in RPMI-1640 with 10% FBS and incubated with viable *E. coli* (1 × 10^5^ CFU; ATCC #23848) for 6 h at 37 °C in 5% CO_2_. As a control, the same number of *E. coli* were incubated without leukocytes in the medium. Serial dilutions of the culture medium were plated on LB agar plates and bacterial colonies were counted after 18 h of incubation at 37 °C.

### 4.6. Quantitative Reverse Transcription-Polymerase Chain Reaction (qRT-PCR)

Total RNA was extracted using an RNeasy Mini Kit (Qiagen, Hilden, Germany) according to the manufacturer’s instructions. cDNA was synthesized from total RNA (500 ng) using the SuperScript III First-Strand Synthesis System (Invitrogen, Carlsbad, CA, USA). qRT-PCR was performed on a LightCycler 480 System (Roche Diagnostics, Mannheim, Germany) using FastStart SYBR Green Master reagent (Roche Diagnostics). Data were calculated using the cycle threshold (^ΔΔ^CT) method and normalized to the expression of ribosomal protein S18 (*Rps18*) for each sample. Primers for SYBR qRT-PCR are listed in Table 1.

### 4.7. Enzyme-Linked Immunosorbent Assay (ELISA)

The concentrations of TNF, IL-6, IFN-γ, and IL-10 in the serum were analyzed using ELISA kits (BD Biosciences) according to the manufacturer’s instructions.

### 4.8. ALT Level Determination

Serum ALT levels were measured using a DRICHEM 3000V instrument (Fuji Medical Systems, Tokyo, Japan).

### 4.9. Statistical Analyses

Statistical analyses were performed using GraphPad Prism 9 (GraphPad Software Inc., La Jolla, CA, USA). Differences between the two experimental groups were evaluated using the Student’s *t*-test or Mann–Whitney *U* test. The time courses and pair-wise comparisons were evaluated using repeated measures two-way analysis of variance with Bonferroni’s post-hoc test. Survival statistics were performed using the Kaplan–Meier method and the log-rank test. Statistical significance was set at *p* < 0.05, with * *p* < 0.05 and ** *p* < 0.01.

## 5. Conclusions

In conclusion, a single treatment with pioglitazone in burn-injured mice modified Kupffer cell function and alleviated uncontrolled inflammatory responses to bacterial challenges, resulting in enhanced survival after infection. Hence, PPARγ activation aimed at strengthening the immune response and immunomodulation could be used as an alternative antimicrobial strategy to avoid bacterial infections in burn patients predisposed to infectious complications.

## Figures and Tables

**Table 1:** The sequence of primers used on the study.

Gene Name	Forwards	Reverse
*MR*	5′-CCCAAGGGCTCTTCTAAAGCA-3′	5′-CGCCGGCACCTATCACA-3′
*Arg1*	5′-CTCCAAGCCAAAGTCCTTAGAG-3′	5′-AGGAGCTGTCATTAGGGACATC-3′
*Ym1*	5′-TCACTTACACACATGAGCAAGAC-3′	5′-CGGTTCTGAGGAGTAGAGACCA-3′
*CRIg*	5′-GTCCTGCAGCGGAACAAGATATAA-3′	5′-GACTTGACCACTAATGGGACTGGAA-3′
*CD68*	5′-CCACAGTTTCTCCCACCACA-3′	5′-AATTTGGGGCTTGGAGCTGAA-3′
*iNOS*	5′-GCAGAGATTGGAGGCCTTGTG-3′	5′-GGGTTGTTGCTGAACTTCCAGTC-3′
*TNF*	5′-CCAGAAAAGACACCATGAGCAC-3′	5′-TCACCCCGAAGTTCAGTAGACA-3′
*Rps18*	5′-TTCTGGCCAACGGTCTAGACAAC-3′	5′-CCAGTGGTCTTGGTGTGCTGA-3′

*MR*: mannose receptor (CD206); *Arg1*: arginase 1; *Ym1*: chitinase-like protein 3 (Chil3); *CRIg*: complement receptor immunoglobulin; *iNOS*: inducible nitric oxide synthase; *TNF*: tumor necrosis factor; *Rps18*: ribosomal protein S18.

## Data Availability

Not applicable.

## Supplementary Materials

The following supporting information can be downloaded at: https://www.mdpi.com/article/10.3390/ijms232112746/s1.

## References

[1] Lachiewicz A.M., Hauck C.G., Weber D.J., Cairns B.A., van Duin D. (2017). Bacterial Infections After Burn Injuries: Impact of Multidrug Resistance. Clin. Infect. Dis..

[2] Jeschke M.G., van Baar M.E., Choudhry M.A., Chung K.K., Gibran N.S., Logsetty S. (2020). Burn injury. Nat. Rev. Dis. Prim..

[3] Belba M.K., Petrela E.Y., Belba A.G. (2017). Epidemiology and outcome analysis of sepsis and organ dysfunction/failure after burns. Burns.

[4] Krishnan P., Frew Q., Green A., Martin R., Dziewulski P. (2013). Cause of death and correlation with autopsy findings in burns patients. Burns.

[5] Zhang P., Zou B., Liou Y.C., Huang C. (2021). The pathogenesis and diagnosis of sepsis post burn injury. Burns Trauma.

[6] Van der Heide D., Weiskirchen R., Bansal R. (2019). Therapeutic Targeting of Hepatic Macrophages for the Treatment of Liver Diseases. Front. Immunol..

[7] Bilzer M., Roggel F., Gerbes A.L. (2006). Role of Kupffer cells in host defense and liver disease. Liver Int..

[8] Dhainaut J.F., Marin N., Mignon A., Vinsonneau C. (2001). Hepatic response to sepsis: Interaction between coagulation and inflammatory processes. Crit. Care Med..

[9] Li P., He K., Li J., Liu Z., Gong J. (2017). The role of Kupffer cells in hepatic diseases. Mol. Immunol..

[10] Inatsu A., Kinoshita M., Nakashima H., Shimizu J., Saitoh D., Tamai S., Seki S. (2009). Novel mechanism of C-reactive protein for enhancing mouse liver innate immunity. Hepatology.

[11] Kinoshita M., Seki S., Ono S., Shinomiya N., Hiraide H. (2004). Paradoxical effect of IL-18 therapy on the severe and mild Escherichia coli infections in burn-injured mice. Ann. Surg..

[12] Miyazaki H., Kinoshita M., Ono S., Nakashima M., Hara E., Ohno H., Seki S., Saitoh D. (2011). Augmented bacterial elimination by Kupffer cells after IL-18 pretreatment via IFN-gamma produced from NK cells in burn-injured mice. Burns.

[13] Xiu F., Jeschke M.G. (2013). Perturbed mononuclear phagocyte system in severely burned and septic patients. Shock.

[14] Croasdell A., Duffney P.F., Kim N., Lacy S.H., Sime P.J., Phipps R.P. (2015). PPARgamma and the Innate Immune System Mediate the Resolution of Inflammation. PPAR Res..

[15] Lehrke M., Lazar M.A. (2005). The many faces of PPARgamma. Cell.

[16] Bouhlel M.A., Derudas B., Rigamonti E., Dievart R., Brozek J., Haulon S., Zawadzki C., Jude B., Torpier G., Marx N. (2007). PPARgamma activation primes human monocytes into alternative M2 macrophages with anti-inflammatory properties. Cell Metab..

[17] Zingarelli B., Cook J.A. (2005). Peroxisome proliferator-activated receptor-gamma is a new therapeutic target in sepsis and inflammation. Shock.

[18] Bedi B., Yuan Z., Joo M., Zughaier S.M., Goldberg J.B., Arbiser J.L., Hart C.M., Sadikot R.T. (2016). Enhanced Clearance of Pseudomonas aeruginosa by Peroxisome Proliferator-Activated Receptor Gamma. Infect. Immun..

[19] Ferreira A.E., Sisti F., Sonego F., Wang S., Filgueiras L.R., Brandt S., Serezani A.P., Du H., Cunha F.Q., Alves-Filho J.C. (2014). PPAR-gamma/IL-10 axis inhibits MyD88 expression and ameliorates murine polymicrobial sepsis. J. Immunol..

[20] Kaplan J., Nowell M., Chima R., Zingarelli B. (2014). Pioglitazone reduces inflammation through inhibition of NF-kappaB in polymicrobial sepsis. Innate Immun..

[21] Liu S.Y., Huang C.C., Huang S.F., Liao T.L., Kuo N.R., Yang Y.Y., Li T.H., Liu C.W., Hou M.C., Lin H.C. (2021). Pioglitazone Ameliorates Acute Endotoxemia-Induced Acute on Chronic Renal Dysfunction in Cirrhotic Ascitic Rats. Cells.

[22] Nakashima M., Kinoshita M., Nakashima H., Kotani A., Ishikiriyama T., Kato S., Hiroi S., Seki S. (2019). Pioglitazone improves phagocytic activity of liver recruited macrophages in elderly mice possibly by promoting glucose catabolism. Innate Immun..

[23] Murray P.J. (2017). Macrophage Polarization. Annu. Rev. Physiol..

[24] Lefere S., Puengel T., Hundertmark J., Penners C., Frank A.K., Guillot A., de Muynck K., Heymann F., Adarbes V., Defrene E. (2020). Differential effects of selective- and pan-PPAR agonists on experimental steatohepatitis and hepatic macrophages. J. Hepatol..

[25] Linares I., Farrokhi K., Echeverri J., Kaths J.M., Kollmann D., Hamar M., Urbanellis P., Ganesh S., Adeyi O.A., Yip P. (2018). PPAR-gamma activation is associated with reduced liver ischemia-reperfusion injury and altered tissue-resident macrophages polarization in a mouse model. PLoS ONE.

[26] Luo W., Xu Q., Wang Q., Wu H., Hua J. (2017). Effect of modulation of PPAR-gamma activity on Kupffer cells M1/M2 polarization in the development of non-alcoholic fatty liver disease. Sci. Rep..

[27] Odegaard J.I., Ricardo-Gonzalez R.R., Goforth M.H., Morel C.R., Subramanian V., Mukundan L., Red Eagle A., Vats D., Brombacher F., Ferrante A.W. (2007). Macrophage-specific PPARgamma controls alternative activation and improves insulin resistance. Nature.

[28] Gao M., Jiang Y., Xiao X., Peng Y., Xiao X., Yang M. (2015). Protective effect of pioglitazone on sepsis-induced intestinal injury in a rodent model. J. Surg. Res..

[29] Kimura T., Nada S., Takegahara N., Okuno T., Nojima S., Kang S., Ito D., Morimoto K., Hosokawa T., Hayama Y. (2016). Polarization of M2 macrophages requires Lamtor1 that integrates cytokine and amino-acid signals. Nat. Commun..

[30] Li Y.H., Zhang Y., Pan G., Xiang L.X., Luo D.C., Shao J.Z. (2022). Occurrences and Functions of Ly6C(hi) and Ly6C(lo) Macrophages in Health and Disease. Front. Immunol..

[31] Ginhoux F., Jung S. (2014). Monocytes and macrophages: Developmental pathways and tissue homeostasis. Nat. Rev. Immunol..

[32] Ikeda N., Asano K., Kikuchi K., Uchida Y., Ikegami H., Takagi R., Yotsumoto S., Shibuya T., Makino-Okamura C., Fukuyama H. (2018). Emergence of immunoregulatory Ym1(+)Ly6C(hi) monocytes during recovery phase of tissue injury. Sci. Immunol..

[33] Wong C.H., Jenne C.N., Petri B., Chrobok N.L., Kubes P. (2013). Nucleation of platelets with blood-borne pathogens on Kupffer cells precedes other innate immunity and contributes to bacterial clearance. Nat. Immunol..

[34] Flannagan R.S., Jaumouille V., Grinstein S. (2012). The cell biology of phagocytosis. Annu. Rev. Pathol..

[35] Helmy K.Y., Katschke K.J., Gorgani N.N., Kljavin N.M., Elliott J.M., Diehl L., Scales S.J., Ghilardi N., van Lookeren Campagne M. (2006). CRIg: A macrophage complement receptor required for phagocytosis of circulating pathogens. Cell.

[36] Liu G., Fu Y., Yosri M., Chen Y., Sun P., Xu J., Zhang M., Sun D., Strickland A.B., Mackey Z.B. (2019). CRIg plays an essential role in intravascular clearance of bloodborne parasites by interacting with complement. Proc. Natl. Acad. Sci. USA.

[37] Gorgani N.N., Thathaisong U., Mukaro V.R., Poungpair O., Tirimacco A., Hii C.S., Ferrante A. (2011). Regulation of CRIg expression and phagocytosis in human macrophages by arachidonate, dexamethasone, and cytokines. Am. J. Pathol..

[38] Gauglitz G.G., Song J., Herndon D.N., Finnerty C.C., Boehning D., Barral J.M., Jeschke M.G. (2008). Characterization of the inflammatory response during acute and post-acute phases after severe burn. Shock.

[39] Flannagan R.S., Cosio G., Grinstein S. (2009). Antimicrobial mechanisms of phagocytes and bacterial evasion strategies. Nat. Rev. Microbiol..

[40] Canton J., Khezri R., Glogauer M., Grinstein S. (2014). Contrasting phagosome pH regulation and maturation in human M1 and M2 macrophages. Mol. Biol. Cell.

[41] Ganeshan K., Chawla A. (2014). Metabolic regulation of immune responses. Annu. Rev. Immunol..

[42] Viola A., Munari F., Sanchez-Rodriguez R., Scolaro T., Castegna A. (2019). The Metabolic Signature of Macrophage Responses. Front. Immunol..

