# Supplementary material for: Pioglitazone Modifies Kupffer Cell Function and Protects against Escherichia coli-Induced Bacteremia in Burned Mice
Source: Int J Mol Sci. 2022 Oct 22;23(21):12746. doi: 10.3390/ijms232112746 (PMC9657905; doi:10.3390/ijms232112746)
Supplement: Supplementary file 1 [file ijms-23-12746-s001.zip › ijms-1945007-supplementary.pdf]

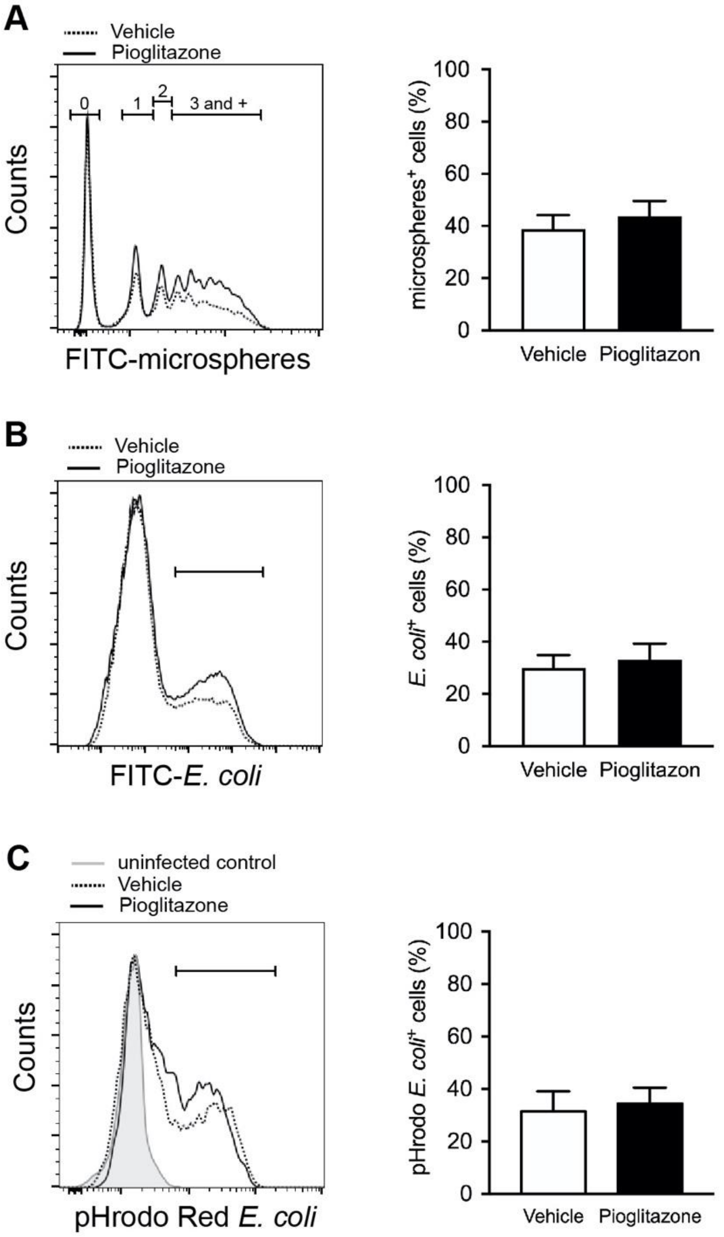

Figure S1. Phagocytosis and bactericidal activity of CD11b$^+$ F4/80^{low} infiltrated monocytes/macrophages in vehicle- and pioglitazone-treated mice after burns. (**A**,**B**) Isolated hepatic immune cells were incubated with fluorescein-conjugated microspheres or *Escherichia coli* for 30 min and stained with anti-CD45, anti-CD11b, and F4/80 antibodies, followed by the analysis of phagocytic activity in F4/80^{low} cells via flow cytometry. (**A**) Representative flow cytometry histogram showing uptake of FITC-microspheres by F4/80^{low} cells. Percentage of F4/80^{low} cells which has internalized ≥ three microspheres was determined. (**B**) Representative histograms showing phagocytosis of FITC-conjugated *E. coli* by F4/80^{low} cells and percentage of FITC-conjugated *E. coli*. (**C**) Representative flow cytometry histogram showing a shift in fluorescence intensity of pHrodo *E. coli* bioparticles in F4/80^{low} cells. Percentage of *E. coli* particle-positive F4/80^{low} cells in vehicle and pioglitazone-treated mice 20 min after administration of pHrodo *E. coli*. n = 6 per group; Data are represented as the mean ± deviation (SD).
